# Prevalence and distribution of selected cervical human papillomavirus types in HIV infected and HIV uninfected women in South Africa, 1989–2021: A narrative review

**DOI:** 10.4102/sajid.v37i1.363

**Published:** 2022-06-08

**Authors:** Rixongile R. Rikhotso, Emma M. Mitchell, Daniel T. Wilson, Aubrey Doede, Nontokozo D. Matume, Pascal O. Bessong

**Affiliations:** 1 Department of Biochemistry and Microbiology, Faculty of Science, Engineering and Agriculture, University of Venda, Thohoyandou, South Africa; 2 Department of Family, Community and Mental Health Systems, School of Nursing, University of Virginia, Charlottesville, United States of America; 3 Claude Moore Health Sciences Library, School of Nursing, University of Virginia, Charlottesville, United States of America; 4 Department of Family Medicine and Public Health, University of California San Diego, California, United States of America; 5 HIV/AIDS & Global Health Research Programme, University of Venda, Thohoyandou, South Africa; 6 Center for Global Health Equity, School of Medicine, University of Virginia, Charlottesville, Virginia, United States of America

**Keywords:** HPV types, HPV prevalence, HPV, HIV, Women, South Africa

## Abstract

**Background:**

Human papillomavirus infection, a causative agent of cervical cancer, is of great concern, more so in populations with high HIV prevalence, such as South Africa.

**Aim:**

This review aimed to examine the prevalence and distribution of selected cervical human papillomavirus (HPV) types in HIV infected and HIV uninfected women in South Africa.

**Methods:**

PubMed and Web of Science databases were searched using key words. For data integrity, data was assessed by two authors independently. The study inclusion criteria comprised records on cervical HPV, HPV genotyping and HPV type distribution among South African women. Statistical analysis was performed using Social Science Statistics.

**Results:**

Sixty-nine articles met the inclusion criteria for analysis. Data on cervical HPV prevalence and type distribution was available only for five of the nine provinces of South Africa. Only 4/69 studies used sequencing as an approach to identify HPV types. In a general population, HPV type 16 was the most frequent (8.80%), followed by types 35 (4.86%), 18 (4.14%), 58 and 52 with the frequency of 3.65% and 3.62%, respectively. Furthermore, the least frequent type was HPV 70 (0.74%). Both HIV infected and HIV uninfected populations had a higher prevalence of high-risk human papillomavirus (hrHPV) types 16, 18 and 35 than other HPV types; while HPV types 6, 11 and 70 were the least frequent types from these populations. Lastly, HPV 16 was the most predominant type among women with normal (2.03%) and abnormal cervical cytology (6.60%).

**Conclusion:**

Expanding on HPV genotyping will improve the knowledge in patterns of HPV type distribution in South Africa that will further help in decision making to improve current diagnostics, and future vaccine development and assessment.

## Introduction

Human papillomavirus (HPV) and human immunodeficiency virus (HIV) are leading sexually transmitted infections (STIs)^1^ that cause significant burden of disease in low- and middle-income countries.^2,3^ Cervical cancer (CC) is underlined as an HIV-associated malignancy and is rated as the second most frequent cancer among South African women of all age groups^4^ after breast cancer. About 70% of CC cases are caused by persistent infection of high risk human papillomavirus (hrHPV) types 16 and 18.^5^ In 2020, there were 10 702 new cases of CC among South African women reported, irrespective of HIV status; of these 5870 women died because of the disease.^6^

Globally, South Africa has the highest HIV infection rates with an estimate of 8.23 million people living with HIV in 2021^7^ with women being the most affected group. However, the introduction and free access to anti-retroviral therapy (ART) has increased the life span of individuals living with HIV and AIDS,^8^ leading to an increasing number of people ageing with HIV infection. Women living with HIV subsequently experience life expectancy approaching that of persons living without HIV, although, they still have an increased incidence of developing CC at a later stage^9^ probably due to their compromised immune system.

According to the International HPV Reference Center, to date there are 228 HPV genotypes that are known,^10^ and about 40 of these genotypes have been reported to infect the ano-genital region.^11^ Based upon the potential interconnection between various HPV genotypes and CC, HPV was thereafter stratified into two main groups: low (lr) and high risk (hr) genotypes primarily known to be non-cancerous^12^ and cancerous,^13^ respectively.

There is currently no cure for HPV infection. However, prevention is possible through vaccination. Three HPV vaccines have been developed thus far, which offers protection against some of the hr types. The global uptake of these vaccines varies geographically.^9^ Cervarix is a bivalent vaccine that covers HPV types 16 and 18; Gardasil is a quadrivalent vaccine offering protection against HPV genotype 6, 11, 16 and 18. Lastly, Gardasil-9 is a nonavalent vaccine and comprises the four HPV types covered by the quadrivalent vaccine, with the addition of types 31, 33, 45, 52, and 58. However, in South Africa, only Cervarix and Gardasil are currently available.

The Department of Health in collaboration with the Department of Basic Education launched a South African HPV vaccination campaign in 2014. The campaign specifically targets 9-year olds and above public-school children in grade 4 and involved delivering two doses of vaccine 6 months apart.^14,15^ In 2014, it was reported that more than 350 000 school girls received the vaccine which covered more than 16 000 of public schools. Within the same year of the programme, 86.6% coverage was attained.^14^ These campaigns and programmes play a role in the reduction of HPV infections and effectively control and prevent CC development in the later stage. However, issues of cost and unavailability of vaccines limit the progress of these programmes as well as the limitation of the available HPV vaccines to cover all the prevalent HPV or hrHPV types.

Immunocompromised individuals, especially HIV-positive women are at high risk of HPV infections^16^ and associated pathologies. Studies have shown a high prevalence of hrHPV types in HIV positive women than negative women^17,18^ and other studies indicate multiple hrHPV infections in HIV positive women.^19,20^ However, other studies indicate a high prevalence of HPV infections in women regardless of HIV status.^19,21^ Therefore, there are uncertainties on the prevalence and distribution of HPV types regarding HIV status, and more studies are needed to understand which hrHPV types are associated with HIV and how they contribute to the development of CC in this population, especially in South Africa where there is a high prevalence of HIV infection.

For these reasons, this review reports on the literature specific to South Africa, highlighting the following: (1) The distribution of cervical HPV studies across provinces in South Africa, (2) The diagnostic methods used to identify HPV types in South Africa, (3) The prevalence and distribution of hrHPV types among women in general, and among those with a normal and abnormal cervical cytology, (4) The prevalence and distribution of hr/lrHPV types by HIV status.

## Review methodology

### Eligibility criteria

Research articles focusing on cervical HPV infection in South Africa were sought. Records on cervical HPV, HPV genotyping and type distribution published among South African women formed part of the study inclusion criteria. Five major exclusion criteria were designated: (1) not geographically related (South Africa must have been at least one of the countries included); (2) review articles, clinical trials and preface; (3) poster presentations, letters to the editor and correspondence; (4) not HPV or cervical (HPV or CC) related and (5) HPV in men. Full text articles published in English were included. Of note, comparative studies conducted between South Africa and other countries were included in the final analysis. However, in such studies only South African data relevant to our review was extracted.

### Information sources

Published reports on the prevalence and cervical HPV types among South African women living with and without HIV were carefully searched and compiled. In addition, articles that did not differentiate HPV prevalence and types by HIV status were also included to answer objectives which were independent of HIV status. All the reports were identified through PubMed and Web of Science databases last consulted on 01 July 2021.

### Search strategy

All the records were sourced using an advanced search option for both databases. In addition, all fields were also searched. The following search strategies were generated per database:

#### PubMed: ((((HPV) OR ((Human Papillomavirus))) OR ((Human Papilloma virus))) OR (Papillomaviridae)) AND ((South Africa*))

(“HPV”[All Fields] OR (“alphapapillomavirus”[MeSH Terms] OR “alphapapillomavirus”[All Fields] OR (“human”[All Fields] AND “papillomavirus”[All Fields]) OR “human papillomavirus”[All Fields]) OR (“papillomaviridae”[MeSH Terms] OR “papillomaviridae”[All Fields] OR (“human”[All Fields] AND “papilloma”[All Fields] AND “virus”[All Fields]) OR “human papilloma virus”[All Fields]) OR (“papillomaviridae”[MeSH Terms] OR “papillomaviridae”[All Fields])) AND (“South”[All Fields] AND “africa*”[All Fields])

### Translations

#### Human papillomavirus

“alphapapillomavirus”[MeSH Terms] OR “alphapapillomavirus” [All Fields] OR (“human”[All Fields] AND “papillomavirus”[All Fields]) OR “human papillomavirus”[All Fields]

#### Human papilloma virus

“papillomaviridae”[MeSH Terms] OR “papillomaviridae”[All Fields] OR (“human”[All Fields] AND “papilloma”[All Fields] AND “virus”[All Fields]) OR “human papilloma virus”[All Fields]

#### Papillomaviridae

“papillomaviridae”[MeSH Terms] OR “papillomaviridae”[All Fields]

#### Web of science

TS=(HPV) OR TS=(Human Papillomavirus) OR TS=(Human Papilloma virus) OR TS=(Papillomaviridae) AND TS=(South Africa)

### Selection process and study risk of bias assessment

The full-text of the relevant articles were read and analysed for the objectives of the current study. Variables required to answer the study objectives were extracted and tabulated into their respective columns. Two authors independently reviewed the search and selection procedure for consistency. Of note, the authors agreed that only articles that strictly speak of the study inclusion criteria be the ones to be considered for further analysis. Variables required that were not reported in certain articles were marked and the authors agreed that such information should be labelled as ‘data was not reported’.

### Data collection process

All records searched from both databases were then exported to Mendeley Desktop, a referencing tool and further processed to remove duplicates and screened for eligibility. The title and abstract were thoroughly screened in Mendeley Desktop and later full articles were downloaded and screened one at a time. This was done by two authors independently using the same tool.

### Data items

**FIGURE 1:**
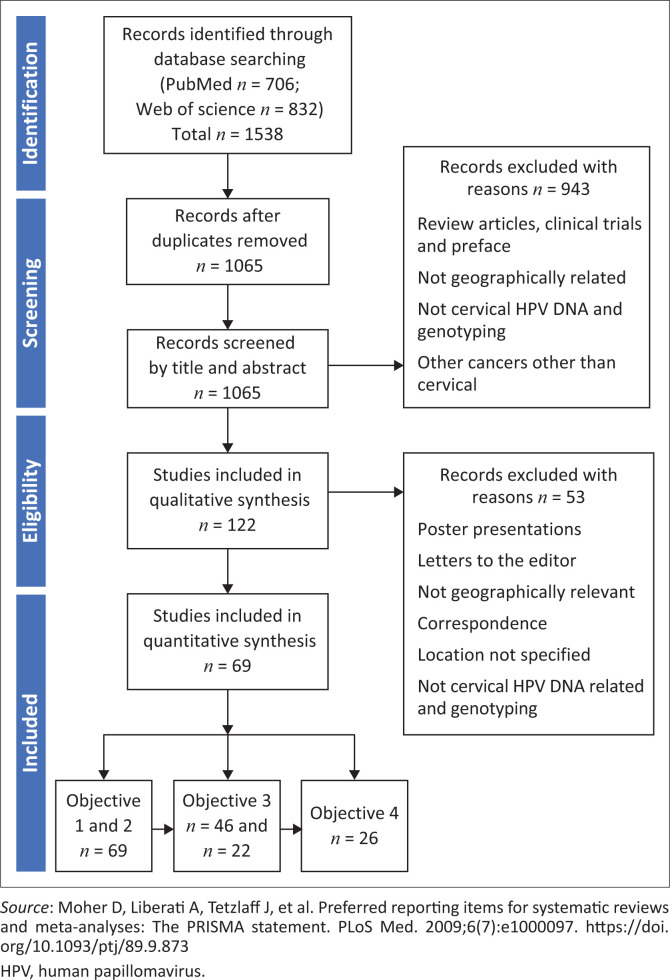
Preferred Reporting Items for Systematic Reviews and Meta-Analyses (PRISMA) workflow for the selection of studies to examine the human papillomavirus types among South African women living with and without human immunodeficiency virus (1989–2021).

Research articles focusing on cervical HPV infection in South Africa published between 1989 and 2021 were retrieved. Data extracted included: (1) relevant information on sample type (cervical cytology/cervicovaginal, biopsies) and from women; (2) sampling device and genotyping technique used; (3) study design, collection time period and age range; (4) study population and sample size; (5) study setting; and (6) data on cervical status. Following the PRISMA protocol,^22^ (Figure 1) a total of 69 published articles were included for final analysis after screening.

### Synthesis method

The full-text of the relevant articles were read and analysed for the objectives of the current study which were grouped into four objectives: (1) The distribution of cervical HPV studies across provinces in South Africa, (2) The diagnostic methods used to identify HPV types in South Africa, (3) The prevalence and distribution of hrHPV types among women in general population and among those with a normal and abnormal cervical cytology, (4) The prevalence and distribution of hr/lrHPV types by HIV status.

To achieve objective 1 and 2, variables required to answer these objectives were extracted from individual records and tabulated into their respective columns using a word file. The variables were (1) author (year), and study title, (2) sample type, sampling device and genotyping technique, and (3) province in South Africa. Of note, these objectives were analysed regardless of the HIV status of study participants recruited, considering all the 69 studies that were included in the quantitative analysis. Records missing certain required information were identified and marked as not reported (Supplementary Table 2).

To achieve objective 3, data was first pooled from 46 of 69 studies to estimate the prevalence and distribution of hrHPV types among women in the general population that is regardless of the HIV status in the country (Supplementary Table 3). To do so, the 69 records were further scrutinised and all the records that (1) reported the distribution of HPV types based on the vaccine coverages, (2) reported HPV types collectively as lr/hr HPV types and/or single/multiple infections instead of reporting on the individual types across the study participants, (3) studies that updated data from the previously published data, (4) studies whose results were showed by graphs making it difficult to interpret the proportions based on the scales used, and (5) comparative studies which did not separate the distribution of the HPV DNA by country, such records were excluded. Of note, to estimate the overall number of study participants, studies that used the same samples such participants were counted once, this was done to avoid over-representation of the population size. Furthermore, a sub-analysis was conducted from 22/46 studies that clearly documented the prevalence of hr-HPV types among South African women according to cervical cytology status.

To achieve objective 4, which was to determine the prevalence and distribution of hr/lrHPV types by HIV status, an intra-search strategy using Mendeley was conducted again within the 46 studies. The following steps were taken. The upper right section of Mendeley page was clicked to help segregate the records by HIV status. Key words such as Human Immunodeficiency Virus, HIV, HIV-infected/positive and HIV-uninfected/negative/HIV non-infected and HIV non-positive were entered into the search box interchangeably to search for HPV records in the library that speaks of HIV status. The resulting 26 records that clearly specified the distribution of HPV types according to HIV status in the country were then grouped into a sub-folder for analysis. Of note, part of the excluded records included all studies that had unreported South African province, unspecified HIV status and those that did not segregate the distribution of HPV types by HIV status.

### Analysis for human papillomavirus types prevalence and distribution in South Africa

Eligible literature was screened by provinces in South Africa that reported for HPV types, types of specimen, laboratory technique used to identity HPV types and HIV status.

Outcomes were tabulated according to author and year of publication, province of study, study population (women living with and without HIV), and study design. Extracted data was examined, across the country, for the prevalence of each of the 14 hrHPV types (16, 18, 31, 33, 35, 39, 45, 51, 52, 56, 58, 59, 68 and 70)^23,24^ regardless of the HIV status first and then classified according to HIV status with two low risk human papillomavirus (lrHPV) type 6 and 11 included.

### Statistical analysis

An online Social Science Statistics tool using option *p*-value calculators was used to derive the *p*-values from the chi square. This was employed in order to determine the association between HIV status and the distribution of HPV infection. A positive significant test was considered statistically significant at *p* < 0.05.

## Review findings

### Characteristics of studies analysed

According to our inclusion criteria, 69 studies were selected for analysis, and represented only five (Western Cape, Eastern Cape, Gauteng, KwaZulu-Natal and Limpopo) of the nine provinces of South Africa. Overall, a total of 38 049 women participated in these studies, of whom, 9955 and 20 747 were living with and without HIV, respectively. Western Cape province had the highest number of study participants (*n* = 27 779) followed by Gauteng province (*n* = 5817), Limpopo province has been observed to have the least number (*n* = 100) of study participants. Of note, to avoid over representation of the study participants, all studies that used the same sample size were counted once. Supplementary Table 1, show the characteristics of all the 69 articles included in the quantitative analysis.

Fifty two of the 69 studies were conducted uniquely in Western Cape (*n* = 31), Gauteng (*n* = 12), Eastern Cape (*n* = 5), KwaZulu-Natal (*n* = 3), and Limpopo (*n* = 1). In two studies, participants were recruited from more than one province: one study comprised participants from KwaZulu-Natal and Gauteng, and the other had participants from Western Cape and Gauteng provinces. In two studies, the province from which participants were recruited was not indicated. Furthermore, 13 comparative studies were conducted between South Africa and other countries: three studies were reported for Britain and South Africa (KwaZulu-Natal), seven studies reported for Burkina Faso and South Africa (Gauteng), and one study was conducted in three countries – Ghana, Nigeria, and South Africa. Lastly, one study each was conducted in Limpopo and Tanzania while the other one in KwaZulu-Natal and Nairobi. These studies are represented in Supplementary Table 2.

The 69 studies eligible for analysis comprised three study populations: HIV non-infected (*n* = 6), HIV-positive (*n* = 17), and both HIV positive and non-infected populations (*n* = 31). Fifteen studies had unspecified HIV status. Overall, there were 48 studies that included HIV-positive populations, including those that comprised both HIV-positive and HIV uninfected individuals.

### Sample type, collection mode, and human papillomavirus genotyping approaches

Of the 69 studies analysed, we grouped specimens into cervical/cervicovaginal lavages, biopsies, self-collected and both self-collected/cervical specimens. The most commonly used specimens included cervical/cervicovaginal lavages, reported in 40 studies, and cervical biopsies, reported in 17 studies. In addition, in six studies specimens were both cervical and self-collected, while in six studies self-collected specimens were used. Of the devices and tools used to collect these specimens, brushes were the commonly used tool reported in 32 of the 69 studies. The use of a spatula was among the least used collection device, Supplementary Table 2.

There is a fair degree of heterogeneity on the approaches used to genotype HPV. Linear Array (LA) was the most commonly used method reported in 30 studies. This was followed by INNOLiPA (*n* = 6) and hybrid capture 2 (HC2) *n* = 4. Enzyme immuno assay (EIA), non-isotopic *in situ* hybridisation (NISH), and Xpert HPV were used in three studies each, while southern blot hybridisation was applied in two studies. HPV direct flow chip and TaqMan based quantification polymerase chain reaction (PCR) was applied once, respectively. Of the studies that used more than one technique, HC2/LA was the most commonly used (*n* = 4), followed by HC2/INNO Lipa (*n* = 2). The least frequently used technique included LA/Xpert HPV, care HPV/INNO Lipa, reverse line blot/PreTeck HPV proofer, HC1/HC2, HC2/hpVIR, and cobas test/LA/HC2 which were used in one study each. In one comparative study, Illumina detected 16 HPV types, including type 30, 74, 86 and 90, not included in LA; and Sanger sequencing was seen to resolve HPV DNA (Deoxyribonucleic acid) untypable by Restriction fragment length polymorphism (RFLP) in another study. Lastly, one study used southern blot hybridisation and sequencing, while the other study compared the genotypic outcomes obtained through massive parallel sequencing to HPVIR assay LA HPV genotyping test (Supplementary Table 2).

### High risk human papillomavirus type diversity and human immunodeficiency virus status

Firstly, the proportion of 14 hrHPV types was examined, irrespective of the HIV status of the women. Overall, 46 studies were thereafter examined, for the 14 hrHPV types. Of a total of 30 677 participants, HPV type 16 was the most frequent (8.80%), followed by types 35 (4.86%), 18 (4.14%), 58 and 52 with the frequency of 3.65% and 3.62% individually. The least frequent was type 70 (0.74%), (Supplementary Table 3).

Furthermore, a sub-analysis of cytology data demonstrated that a total of 13 044 and 15 782 women had a normal and abnormal pap smear results, respectively. Women with abnormal pap smear had higher prevalences of HPV types when compared to their counterparts. Among women who had an abnormal pap smear, HPV types 16 (6.60%), 35 (2.43%), 18 (2.24%), 58 (1.70%), 33 (1.62%), 45 (1.57% each) and 52 (1.52%) were the seven most prevalent types in descending order of frequency. On the contrary, HPV types 16 (2.03%), 35 (1.85%), 58 (1.58%), 45 (1.55%), 18 (1.40%), 52 (1.30%) and 68 (1.19%) were the most predominant types among women who had a normal pap smear test.

From the 26 studies that clearly documented the proportion of HPV type distribution according to HIV status, these studies were examined to estimate the frequency of 14 hrHPV and 2 lrHPV types and to determine the statistical association between HPV type distribution and HIV status, also considering the types included in Gardasil-9 vaccine. The findings revealed that, similar HPV types were detected in both HIV-positive and HIV-uninfected women, with HPV 16, 18 and 35 being the most common detected types in both HIV-positive and HIV-uninfected populations in the country; however, the proportions varied (Supplementary Table 4). For these types, the proportions observed were as follows: 0.135% and 0.047% (HPV 16); 0.065% and 0.028% (HPV 35); 0.068% and 0.024% for type 18 detected in women living with and without HIV, respectively. On the contrary, HPV 6, 11 and 70 were the least common types with the proportions ranging from 0.001% to 0.015%. Furthermore, statistical analysis depicted that types 6, 11, 16, 18, 31, 33, 35, 39, 45, 51, 52, 56, 58, 68 and 70 were significantly more common among women living with HIV than those who were living without HIV, while type 59 was the only type observed to be significantly more common among women living without HIV. All these HPV types were statistically significant at *p* < 0.00001 (Supplementary Table 4).

## Implications, recommendations and conclusion

This review provides insights on the scope of HPV research on viral diversity and HIV status in South African women between 1989 and 2021. Primarily, it reveals that HPV research is limited to five (Gauteng, KwaZulu-Natal, Limpopo, Western Cape and Eastern Cape) of the nine provinces of the country, and even in this regard, there are relatively few studies from each of the five provinces. Nevertheless, Western Cape province was observed to have the highest number of studies among the five provinces. Factors such as poor access of primary healthcare systems, lack of viral HPV knowledge, lack of pap testing, misconceptions and conspiracy theories associated with the HPV infection at large could have had an impact on the participation of the participants in the studies. Factors such as financial constraints involved to carry out the HPV diversity research studies, lack of HPV research expertise, difficulties in attaining permission (s) and approval (s) from relevant organisations might have been some of the reasons why other provinces lack data on HPV research.

Studies have shown that the distribution of HPV infection differs by province, technique used to detect it, HIV status of participants, specimen collection method and the age group of participants.^25,26,27,28^ The study findings revealed that there are various methods used to identify HPV types in the country, with Roche LA being the most commonly used technique while sequencing is rarely used. Thus, more consistent methods for sample collection and genotyping procedures are necessary to systematically assess the prevalence of potentially carcinogenic types of HPV.

Despite the higher cost involved in next generation sequencing (NGS),^29^ the genotyping tool has proved to be most specific^30^ and sensitive^31,32^ compared to LA genotyping test kit that only detects the restricted number of 37 HPV types incorporated in the kit and has the possibility of missing mixed and minority infections. Flores-Miramontes et al. in Mexico genotyped HPV using both LA and NGS, detected HPV types 32, 44, 74, 102, and 114 not incorporated in the LA.^32^ In South Africa, specifically Cape-Town, Meiring et al. compared LA and NGS and found that NGS was able to detect four additional HPV types (30, 74, 86 and 90) not covered by the 37 restricted types of the LA.^33^ This also implies that the differences on how the samples are genotyped have a great impact on the genotyping outcomes. Thus, the authors agree that more sensitive technologies such as NGS are significant at least at a research level to be used as a strategy to unravel the full spectrum of HPV types in the South African population. Of interest, recent findings documented by Ardhaoui and colleagues, depicted that NGS was able to detect genotypes of clinical importance that include HPV type 31, 39 and 52 that the reverse line hybridisation genotyping technique failed to detect regardless of the presence of their probes embedded on the membrane.^34^ This raises a significant aspect of presumably under reporting of other HPV types of clinical importance in certain cohorts. Of note, kits validation for accuracy purposes and in the management of the disease can be encouraged.

Despite the inconsistencies used in data reporting strategies across the studies, the overall HPV type distribution according to HIV status and in a general population depict that HPV 16, 35 and 18 were the most frequent types in these populations; thus, encourages the need for routine hospital or clinic visits for regular check-ups among South African women, especially those who never received any vaccine. With evidence pointing to a significant increase of HPV more in women living with HIV^16,35,36,37,38^ and in the general population. The findings of the literature review conducted in 2021 by Kombe and colleagues highlighted that, the prevalence of HPV type distribution do vary across the world although that does not change the associated diseases caused by these viral types. Furthermore, there are certain HPV types that have been found to be present around the world.^39^ Several review studies conducted across continents including Africa, revealed that hrHPV types such as 16, 18, 35 and 58 among others are the most frequent types^39^ which validates our study findings.

Human papillomavirus/human immunodeficiency virus co-infection among women particularly in developing countries remains one of the principal public health challenges requiring closer intervention. Worldwide, in a general population HPV types 16, 18 and 45 are primarily linked with CC^23^; however, the prevalence of HPV types varies worldwide. In Asia, HPV 52 and 45 are the most prevalent HPV types; in the USA and Europe, HPV types 45 and 33, respectively, are the most common types, and types 35 and 45 are more predominant in Africa,^40,41,42^ all these types were common after HPV 16 and 18 in participants with invasive CC. Both HPV types 35 and 45 are considered hrHPV^23^ for subsequent CC. Although a number of studies show that the bivalent vaccine does offer protection against these types, cross protection vaccines are less reliable compared to type specific and is of a limited duration.^43^ Neither of these are included in the currently available HPV vaccines in South Africa. To enrich our understanding of HPV types, including detection of minority viral populations in South Africa, the application of sanger sequencing or next-generation sequencing is strongly recommended,^44^ at least for research purposes. This is important to identify putative hrHPV types which may not be covered by the current diagnostic approaches and vaccines.

The state of cervical cytology in the studied population plays a significant role in the distribution of HPV types, be it single and/or multiple infections and the overall prevalence. Our findings revealed that HPV types 16, 35, 58, 45, 18, 52 and 68 and types 16, 35, 18, 58, 33, 45 and 52 were the most prevalent types in descending order among women with normal and abnormal pap smear, respectively. This was in accordance with a study conducted by Wolday and colleagues on the distribution of HPV genotype among Ethiopian women with normal and abnormal cervical cytology. Their findings revealed that HPV types 16, 35, 45, and 18 were among the most prevalent HPV types among women with normal cytology while most women with abnormal cervical cytology had HPV types 16, 45, 31 and 35.^45^ The findings of another study conducted in Turkey by Muderris and colleagues agree with that of our study findings.^46^ In both studies HPV type 16 was the most detected type in both compartments. A meta-analysis data by Obeid et al.^47^ demonstrated that HPV 16 and 58 were the most common types among women with abnormal cervical cytology in the Middle East and North Africa. Of note, we noticed that a normal cervical cytology test does not necessarily mean the absence of hrHPV of clinical importance. Thus, the presence of hrHPV types among women with normal cytology raises a serious challenge among policymakers in South Africa. The study findings encourage that more closer routine check-ups and HPV screening should be prioritised among South African women.

Cervical cancer is responsible for significant mortality among women.^32^ Cervical cancer mortality among women living with HIV infection is twice greater than those living without HIV.^48,49^ Nonetheless, the availability of ART increases the life span of women living with HIV and raises a necessity for the prevention of CC in developing countries.^50^ To fully understand the distribution of HPV types in a specific geographic location, genotyping samples from both individuals living with HIV and without HIV is highly recommended. In the absence of effective screening, CC might kill millions of women.^51^ Human papillomavirus genotyping can be both a secondary prevention technique employed to help in the screening and early detection of CC, as well as a tertiary prevention technique that also helps in the management of pre-cancerous cervical lesions.^52^ In addition, HPV genotyping technique can be used to further investigate women who test positive during cytology.^53^ Possible recurrence of HPV infections can also be monitored using this method during patient follow-ups after their treatment of cervical intraepithelial neoplasia (CIN).^54,55^ Thus, understanding the facet of HPV types would aid in the better strategies of prevention and management of CC.^52^ Despite the late HPV vaccination rollout in South Africa, Western Cape, Gauteng and KwaZulu-Natal provinces do have data depicting the vaccination coverages of first doses among the primary schools ranging between 64.0% and 99.7%.^56,57,58^ However, to the best of our knowledge, checking when the vaccination rollout programme commenced in the country and the target age groups, our study participants analysed are highly unlikely to have been vaccinated or benefited from these programmes. Therefore, perhaps some of the circulating types of clinical significance might have been prevented or lowered provided some of the study participants benefited from the vaccine.

This narrative review should be considered with some limitations. It was not possible to look at the differences in the distribution of HPV types according to socio-economic and vaccine status of the participants due to the lack of data in the analysed studies. It has been demonstrated that these two factors impact the carriage and type of HPV.^59,60^ Secondly, it would have been of interest to review the distribution of hrHPV in women on antiretroviral therapy. Recent data suggests that ART may lead to clearance of HPV infection.^61,62^ Lastly with South Africa having high levels of disparities in terms of access to health and health outcomes from rural and urban settings, it would have been great to determine the HPV type distribution and prevalence in those two settings. However, out of 69^63,64,65,66,67,68,69,70,71,72,73,74,75,76,77,78,79,80,81,82,83,84,85,86,87,88,89,90,91,92,93,94,95,96,97,98,99,100,101,102,103,104,105,106,107,108,109,110,111,112,113,114,115,116,117,118,119,120,121,122,123,124,125^ studies that met our inclusion criteria only six (9%) were conducted specifically in rural areas. Thus, a comparison would be biased towards where more studies were done.

In conclusion, this review describes the prevalence and distribution of cervical HPV types as reported in the literature which only represents participants recruited from five of nine provinces in South Africa, with Roche LA being the commonly used genotyping technique. This raises a potentially serious concern due to the lack of research studies and data in the country. Furthermore, we observed that hrHPV type 16 was the most predominant type from all the populations analysed. Therefore, further research to expand the scope of HPV types is proposed, including the use of sensitive approaches such as NGS. This will augment the understanding of the patterns of HPV geo-diversity in South Africa, that will further help in decision making with regard to study vaccines and screening strategies that are geographically relevant.
