# Erratum: Prevalence and distribution of selected cervical human papillomavirus types in HIV infected and HIV uninfected women in South Africa, 1989–2021: A narrative review

**DOI:** 10.4102/sajid.v39i1.575

**Published:** 2024-01-30

**Authors:** Rixongile R. Rikhotso, Emma M. Mitchell, Daniel T. Wilson, Aubrey Doede, Nontokozo D. Matume, Pascal O. Bessong

**Affiliations:** 1 Department of Biochemistry and Microbiology, Faculty of Science, Engineering and Agriculture, University of Venda, Thohoyandou, South Africa; 2 Department of Family, Community and Mental Health Systems, School of Nursing, University of Virginia, Charlottesville, United States of America; 3 Claude Moore Health Sciences Library, School of Nursing, University of Virginia, Charlottesville, United States of America; 4 Department of Family Medicine and Public Health, University of California San Diego, California, United States of America; 5 HIV/AIDS & Global Health Research Programme, University of Venda, Thohoyandou, South Africa; 6 Center for Global Health Equity, School of Medicine, University of Virginia, Charlottesville, Virginia, United States of America

In the published article, Rikhotso RR, Mitchell EM, Wilson DT, Doede A, Matume ND, Bessong PO. Prevalence and distribution of selected cervical human papillomavirus types in HIV infected and HIV uninfected women in South Africa, 1989–2021: A narrative review. S Afr J Infect Dis. 2022;37(1), a363. https://doi.org/10.4102/sajid.v37i1.363, an omission error occurred in Online Appendix 1 (supplementary data). Table 2, Table 3 and Table 4, were initially omitted from Online Appendix 1 on the first publication. The updated and correct Online Appendix 1 showing all four tables replaced the incorrect version with its republication.

The publisher apologises for this error. The correction does not change the study’s findings of significance, the overall interpretation of the study’s results, or the scientific conclusions of the article in any way.

